# The Prescription Druggist an Anachronism

**Published:** 1892-02

**Authors:** 


### EDITORIAL DEPARTMENT.

F. E. DANIEL, M. D., EDITOR AND PUBLISHER.

THIS JOURNAL, by unanimous vote of the members, was made the Official Organ of the Austin District Medical Society.

COLLABORATORS:

*Dr. R. M. Swearingen, Austin.*
*Prof. B. E. Hadra, M. D., Galveston.*
*Prof. Geo. Cupples, M. D., San Antonio.*
*Dr. T. C. Osborn, Cleburne, Texas.*
*Dr E. J. Doering, Chicago.*
*Dr. E. J. Beall, Fort Worth.*
*Dr Odo Betz, Germany.*
*Prof. W. B. Rogers, M. D., Memphis.*
*Dr. J. W. McLaughlin, Austin.*
*Dr. T. J. Tyner, Austin.*
*Prof. J. F. Y. Paine, M. D., Galveston.*
*Dr. R. H. L. Bibb, Mexico.*
*Dr. T. J. Bennett. Austin.*
*Dr. Bat Smith, Wharton.*
*Dr. E. Meierhof, New York.*
*L. H. Luce, M. D., West Tisbury, Mass.*

### THE PRESCRIPTION DRUGGIST AN ANACRONISM.

The time has came when the physician should cut loose from the retail druggist, sever all relations with him, and especially that of dependence upon him to compound his prescriptions.

There are many reasons for this. In the first place the average druggist is not the physician's friend in any sense, not, as has always been claimed, his ally or indispensable aid. On the contrary he is too often the enemy of the physician's best interest, and frequently a wolf in sheep's clothing; he plays two games. It is to his interest, the druggist's, to get the physician's patronage, fill his prescriptions, and hence he keeps a pleasant face to him, and endeavors to propitiate him; while at the same time his shelves are full of patent medicines and proprietary medicines, and often, of his own proprietary articles—such as "liver medicines," "anti-bilious" medicines, corn salves, cosmetics, etc., which he does not lose an opportunity to recommend as "good for" such and such complaints; and it has happened that when a man goes into a drug store in quest of a doctor, he has been sent away without seeing the doctor, with a bottle of something which the druggist has sold him with the assurance that it is "good for" his ailment.

No one will question the statement that the prosperity of the patent medicine trade is at the expense of the physician's legitimate work. The medical profession should by every means at their command, oppose the sale of patent medicines, even to the extent of boycotting if necessary, the druggist who handles them.

This is not all; the druggist, as is well known, will not hesitate in the absence of the doctor to prescribe for a patient himself; most of them do it.

Leaving out of consideration the great objection, that when a patient has recovered from a spell of sickness, he has a drug bill to pay,—often as large as the doctor's bill; leaving out of consideration the objection that, no matter how urgent a case may be, a prescription has to be carried to the drug store to be filled, day or night, the messenger has to await his turn,—sometimes hours;—and omitting all consideration of danger of a mistake of the druggist in compounding the medicines, there are abundant other reasons why the system of prescribing should be abandoned.

The above are enough, within themselves; but, in addition, the medicines are often nauseous and disagreeable; the average doctor orders at each prescription usually twenty to forty doses, and perhaps at the very next visit will find it necessary to change the treatment; and after a spell of sickness in a family, the shelf is full of bottles and boxes of medicines, half full, or scarcely touched, all of which are to be, or have been, paid for.

Having prescriptions filled by the druggist is a relic of the dark ages. Formerly, before chemistry had separated the active principles of drugs, the doctor himself compounded his crude drugs; later he had an apothecary to do it for him. The time has come when a physician can carry in a vest pocket, in compact form and neat appearance, all the medicine necessary for a day's or week's use, and in a tasteless form. The progress in pharmacy has been such that the essential part of nearly every drug can now be had in minute granules or tablets, and those of liquid character, in drop doses; and not only this, the enterprising manufacturer keeps up with the literature of medicine, and when he sees that the clinicians and practitioners eminent in the service, find such and such combinations useful in such and such conditions, forthwith the combination is gotten up in a pretty little red or white granule or tablet, ready for use, whether it be by the mouth or by hypodermic injections, and there is no danger of an overdose or of a mistake; the doses are accurately measured, and when it comes to those alkaloids, the dose of which is the hundredth of a grain, this is a desideratum.

Why not carry such medicines? They are inexpensive; and even were they not, the doctor could add a trifle to his bill for medicine furnished. Let every reader of this reflect a moment on this subject. The payment of a big drug bill is avoided, one of the principal reasons some even wealthy people give for not sending for a physician so long as it can be avoided. The delay incidental to the trip to the drugstore, and if at night, rousing the clerk, and of waiting to fill the prescription; the danger of mistake; these are obviated, as well as the great wast e of medicine, which every doctor knows is a result of the prescribing custom. The time lost in this way before the patient can begin to get the benefit of the physician's skill, may be fatal.

But, in case of poor people; it may be that there is no money in the house with which to pay for the medicine at the time,—and "medicines are cash,'' the polite druggist will tell even the little ragged or barefooted girl, who tremblingly hands in the prescription when her turn has come,—and this embarrassment, perhaps distress, can be obviated by what we propose,—the doctor carrying with him such drugs and medicines, and appliances, as he is likely to need. Why, it is a common thing for a messenger to have to come several miles to town after the visit of the doctor to the country, to have a prescription filled.

In brief, there is every reason why the druggist, the prescription druggist, should be relegated with other out-of-date things to the past. He is a middle man, as useless as a fifth wheel to a wagon, and too often a hypocrite to the doctor, a shark to the patient, and an enemy to legitimate practice of medicine.

Let him confine himself to the sale of patent medicines if he will, and come out openly as the opponent and enemy of the medical profession. Let him sell paints and oils and varnish, glass and putty and wall paper, but no longer put up prescriptions for your patients.

We insist that the prescription druggist is an anachronism, and should be laid on the shelf with the lancet, the old squirt syringe, and calomel and jalap powders; he is more; he is very often—not always, however, a shark, and a secret enemy to legitimate medicine. Fortunately there are notable and noble exceptions.

But what shall be said of him who, having ''stolen the livery of heaven to serve the devil in,'' ''stands in'' with the druggist, and actually accepts in money, a percentage on his prescriptions? Nothing! The vocabulary of polite language does not contain words capable of defining him; and he had best be left to his conscience, if he have any, and a hereafter.

It is gratifying to know that many leading physicians are now carrying their medicines, and the practice will soon become general. The *raison de etre* of the prescription druggist has passed away with the progress of the science of pharmacy. He belongs to a past generation.

