# Erratum

**Published:** 1892-02

**Authors:** 


ERRATUM.—In the leading editorial the "H" has accidentally been left out of "anachronism."

